# Pedicled Abdominal Flap in the Youngest Patient Yet? A Case Report of a Newborn with Neonatal Compartment Syndrome

**DOI:** 10.1055/s-0044-1801788

**Published:** 2025-01-17

**Authors:** S. Raja Sabapathy, A. Dharanipriya, Monusha Mohan, Subramanian Ramani, M. Selvaraj, R. Raja Shanmuga Krishnan

**Affiliations:** 1 Department of Plastic, Hand and Reconstructive Microsurgery, Ganga Hospital, Coimbatore, Tamil Nadu, India; 2 Department of Paediatrics and Neonatology, Ganga Women, Child & Speciality Centre, Coimbatore, Tamil Nadu, India

**Keywords:** neonatal compartment syndrome, fasciotomy, pedicled flap, children, abdominal flap

## Abstract

Pedicled abdominal flaps continue to be popular in most parts of the world for covering soft tissue defects of the upper limb. There is apprehension if distant pedicled flaps can be used in children for fear of disruption. We recently had a newborn baby with neonatal compartment syndrome (NCS) of her left upper limb in whom a pedicled abdominal flap was successfully used to cover the raw area in the forearm at 41 days of life. A severely swollen limb with ischemic skin lesions associated with lack of motion of the upper limb often points to NCS. Since no guidelines exist for the diagnosis and management of NCS, a high index of suspicion and urgent fasciotomy are required to limit its sequelae. Our patient had an emergency fasciotomy elsewhere was referred to us with a precariously viable limb for salvage. The raw area in the forearm with exposed bone was successfully covered with a pedicled abdominal flap at 41 days of life. Our patient is probably the youngest patient ever to receive a pedicled abdominal flap.

## Introduction

Soft tissue cover in an infant can be demanding in terms of both surgical skills and pediatric anatomic and physiological challenges. Microvascular procedures like free flap cover can be challenging though not impossible. In complex hand defects especially where the limbs survive on collateral circulation, pedicled flaps come in handy. We report the case of a newborn baby with neonatal compartment syndrome (NCS) and describe the clinical presentation, disease progression, and management. Skin loss, bluish discoloration of digits, necrotic eschar, and swelling in the forearm at birth can be indicators of NCS. NCS demands prompt surgical intervention. 1 2 3 An emergency decompressive fasciotomy should be performed. 1 2 Possible etiology includes intrinsic (coagulation disorders or septicemia) or extrinsic (external compression due to oligohydramnios/fetal posture/cord loops/constriction rings) causes that lead to an increased compartment pressure. 4 The child was diagnosed to have NCS and underwent emergency fasciotomy elsewhere. She was referred to us for the management of the precariously viable limb and for the soft tissue cover. A pedicled abdominal flap was successfully used to cover the forearm defect in the 41-day old baby.

## Case Report

**Fig. 1:**
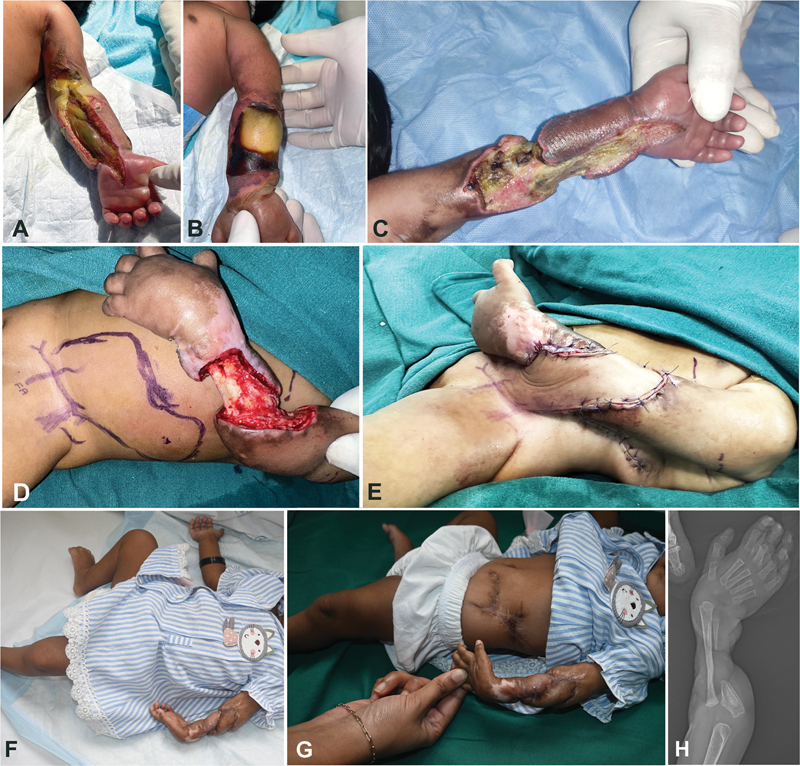
Newborn baby with neonatal compartment syndrome. ( **A, B** ) Fasciotomy wound on the flexor aspect of the left forearm with underlying necrosed flexor muscles. The extensor skin was gangrenous. ( **C** ) The forearm wound after debridement. The hand was surviving on collateral circulation. ( **D** ) Abdominal flap planning and inset. Markings for femoral artery and superficial inferior epigastric artery (SIEA). ( **E** ) Inset of the hypogastric flap on the debrided near-circumferential forearm defect with exposed ulna. ( **F, G** ) The well-healed flap at 5 months of follow-up. The donor site has healed well. ( **H** ) Postoperative X-ray showing the gap in the ulna after sequestrectomy.

A preterm baby girl, born to parents of nonconsanguineous marriage via vaginal delivery, weighing 3 kg, had a swollen left forearm with discolored skin. Except for gestational diabetes, maternal pedal edema, and advanced parental age at conception (maternal age, 37 years and paternal age, 48 years), the antenatal period was uneventful. The pediatric surgeon suspected NCS and performed a fasciotomy at 2 days and 14 hours after birth ( Fig. 1A, B ). The baby was referred to our center when she was 6 days old. There was a near circumferential raw area in the forearm with necrosed flexor and extensor compartments with no distal pulse and a precariously viable limb. The wound was dressed on alternate days and careful serial debridement was done in order not to disturb the collaterals and risk limb loss ( Fig. 1C ). A free flap would have required a long segment of vein graft, as the proximal arterial end would be high up in the arm. Hence, a pedicled abdominal flap was planned ( Fig. 1D, E ). The flap cover was done when the baby was 41 days old and divided after a delay at 21 days, and the division was complete 6 days later. The delay was used to recruit an additional area of flap. Simple adhesive plaster strapping was used. Though the wounds healed well, the forearm developed a discharging sinus secondary to the ulna sequestrum. Sequestrectomy was done 10 days after flap division. At 5 months, the limb wounds had healed but the child had wrist flexion contracture ( Fig. 1F–H ). The flaccid limb with a one bone forearm and an insensate hand would need staged reconstructive procedures later.

## Discussion

Compartment syndrome in a newborn is a rare entity. A recent review reported that only 43 reports describing 86 cases have been published in the period from 1980 to 2021. 1 Our patient had typical features like sentinel skin lesions (bluish digits) and a swollen flaccid limb at birth. 1 2 The baby was delivered preterm and the mother had gestational diabetes. These are the common extrinsic variables reported as potential risk factors for NCS. 1 4 NCS is likely due to events that occur around the delivery of the baby as any skin wound in the fetus would have healed well by delivery, due to the scarless fetal wound healing process. 2 Clinical diagnosis is made if the limb shows swelling, motor weakness, and any two of the sentinel skin lesions like bluish plaque or necrotic eschar or ulceration or blisters. 1

A high degree of suspicion is required for timely intervention. 1 5 Emergency fasciotomy is needed to relieve the high compartment pressure in the forearm and should include carpal tunnel and Guyon canal release. 1 “Time is muscle” holds true in treating acute compartment syndrome. Nerves and muscles cannot tolerate ischemia of more than 6 hours. 6 It is reported that if fasciotomy is performed before 24 hours of birth, there will be less incidence of sequelae like motor impairment, bone growth abnormalities, and risk of amputation. 1 Early diagnosis and urgent fasciotomy are the key.

Postfasciotomy, the salvaged limb needed debridement and soft tissue coverage. A pedicled abdominal flap was necessary in our patient. We have reported the use of a pedicled abdominal flap in an infant and multiple abdominal flaps in an 18-month-old child. 7 8 Al-Qattan and Al-Qattan reported its use in a 20-month-old child. 9 We need to restrain the children when they come out of anesthesia, but they tend to do well once they are awake. Typically, these children are discharged within 4 to 5 days after surgery. They do not tend to pull away the pedicled flaps since doing so would cause them pain.

In NCS, where the affected limb relies on collateral circulation, pedicled flaps are a safer solution since there is negligible proximal dissection to access recipient vessels needed for a free flap. In our patient, a free flap would be challenging as the recipient vessel in the limb was located much more proximally in the arm, requiring long vein grafts, and the size of the defect was much bigger than what is typically repaired with the most commonly used free flaps. A pedicled abdominal flap, in these situations, is a safe and reliable option. 7 10

A newborn with a swollen limb, sentinel skin lesion, and flaccid paralysis should be suspected to have NCS. Emergency fasciotomy as early as possible is recommended. The skin lesion present at birth should not be taken lightly and may be the only early indication of NCS. Pedicled abdominal flaps can safely be done in such young children.
